# Using secure WhatsApp-based digital recruitment as an intervention in prostate cancer research with black men

**DOI:** 10.3389/fdgth.2026.1786070

**Published:** 2026-10-28

**Authors:** Kai Wakeham, George Theodorakopoulos, Shancang Li, Yifan Liu, Xueyi Wang, Nicholas Courtier, Harriet Quinn-Scoggins, Sarah Fry

**Affiliations:** 1 School of Healthcare Sciences, Cardiff University, Cardiff, United Kingdom; 2 School of Computer Science, Cardiff University, Cardiff, United Kingdom; 3 PRIME Centre Wales, Division of Population Medicine, School of Medicine, College of Biomedical and Life Sciences, Cardiff University, Cardiff, United Kingdom

**Keywords:** black men, community engagment, data security, digital health, privacy-by-design, prostate cancer, WhatsApp

## Abstract

This paper describes the development and implementation of a secure, community-centred digital system as an intervention to share prostate cancer risk information among Black men in southeast Wales. In this study, the term Black men is used to refer to men of Somali and African Caribbean heritage. The system was designed to support culturally and linguistically appropriate participation. Recruitment was facilitated by two community co-applicants using project-funded smartphones and WhatsApp Business accounts to disseminate co-created prostate cancer risk messages, link to external prostate cancer risk checker and link to an evaluation survey, within closed community groups. Participants accessed the survey through a custom landing page that verified mobile numbers via CAPTCHA and unique access codes distributed using TextGlobal. After verification, participants selected their preferred language (English or Somali) to view participant information sheets and consent forms. Submitted consent data were encrypted and securely stored on university servers, with cross-referenced verification ensuring integrity. These measures upheld core digital security principles: protecting access to the WhatsApp groups to invited participants, controlling survey access, protecting the confidentiality and accuracy of consent data through encryption, and maintaining secure storage. A privacy risk assessment using the LINDDUN GO framework identified and mitigated vulnerabilities, including risks of data disclosure, unnecessary processing, and regulatory non-compliance. This paper presents a replicable approach for integrating community engagement with privacy-by-design principles in digital health research. It demonstrates how participatory digital systems can enhance inclusivity and trust while upholding data protection, offering practical guidance for researchers designing secure, culturally informed digital methodologies.

## 1 Introduction

Prostate cancer (PCa) is a frequently occurring cancer (1) and is a leading cause of cancer-related death in men (2). PCa is a common cancer in men aged over 70 years, although black men and men with a family history of PCa are at the highest risk of all men (3). The reasons for the increased incidence in black men remain unclear, although recent evidence suggests that, compared to other ethnicities, black men without PCa have higher levels of prostate-specific antigen (PSA), a protein secreted by the prostate that can indicate prostate disease (benign or malignant) (4). This elevated PSA could contribute to overdiagnosis in this group, defined as the detection of cancers that may never become clinically significant, as well as men experiencing unpleasant and occasionally harmful effects of a prostate biopsy (3). Despite this, the burden of PCa is greater amongst black men who present at a younger age and with late stage and higher-grade disease relative to other ethnicities (5). This diagnostic discrepancy is thought to be related to the intersecting influences of genetics, social deprivation, individual capability to engage with healthcare services, and lifestyle (3, 7, 8). Social deprivation is associated with poorer health outcomes, driven by socioeconomic factors that can increase engagement in health-risk behaviours and contribute to delays in cancer diagnosis (6). Barriers to timely diagnosis may include lower levels of health literacy, competing life demands that affect attendance at healthcare appointments, and limited access to services (7, 8). In some cultural groups, stigma associated with this male-specific disease may also affect engagement with healthcare (9). Collectively, these factors can contribute to inequalities in cancer outcomes.

Addressing the challenges of early PCa diagnosis remains a priority. At present, there is no population-based screening for PCa in the United Kingdom (UK), and in November 2025 the UK National Screening Committee published a draft recommendation proposing targeted screening for men with confirmed BRCA1 or BRCA2 gene variants, while further research continues to identify safe and effective screening approaches (10, 11). Given the persistent underrepresentation of Black men in PCa research, and therefore limited representation of this cultural group in research findings (12), there is a clear need for alternative strategies to support both disease awareness-raising and equitable participation in research.

Previous research has shown that men in these communities’ value community-based methods of sharing health information (7, 13, 14) and that using broad terms such as Black Minority Ethnic (BME), Black Asian Minority Ethnic (BAME), or Black obscures important cultural and linguistic differences between groups. In the context of PCa specifically, notable differences have been identified between African-Caribbean and Somali men in how they engage with and share PCa health information (15). In response to these findings, two separate research involvement groups, comprising African Caribbean and Somali men, met in 2023 to explore meaningful ways of sharing PCa risk information within their communities (16). Both groups identified WhatsApp as their most trusted instant messaging platform and reported feeling confident using it to share PCa risk information and culturally relevant messages within their peer networks (Figure 1) (17). The use of WhatsApp for disseminating health information has increased in recent years, due to its low cost and widespread accessibility (18). Additionally, WhatsApp is widely used within many minority communities, because of population mobility and the platform's capacity to facilitate transnational communication (19). These characteristics made WhatsApp particularly relevant to the barriers identified above, as it provided a familiar and accessible channel through which prostate cancer risk information could be shared within established community networks. This paper focuses specifically on the study's digital security framework, describing the processes used to monitor and manage digital security throughout the intervention, including governance arrangements, information-sharing procedures, and measures implemented to protect participant privacy and maintain platform security.

**Figure 1:**
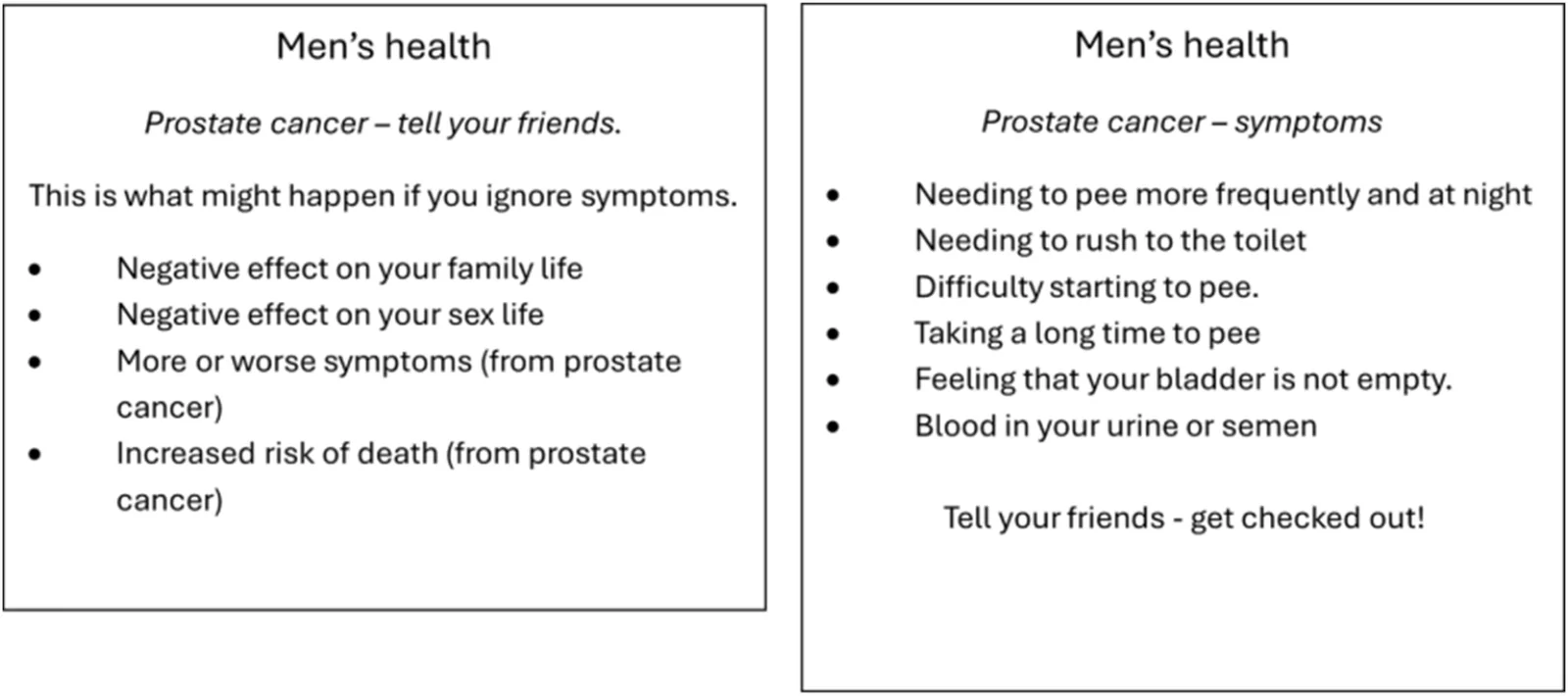
Prostate cancer risk and symptom messages disseminated via WhatsApp (in bold). Risk and symptom messages were co-created with community partners. Symptom messages were based on National Health Service publicly available prostate cancer symptom information (31).

## 2 Materials and methods

### 2.1 Study design

This community-engaged mixed-methods pilot study explored the acceptability and accessibility of peer-led WhatsApp groups for sharing prostate cancer risk messages within Black communities. The study design was informed by Sekhon's Theoretical Framework of Acceptability (TFA) (20),, supported by a privacy risk assessment employing the LINDDUN GO framework (21) to systematically identify privacy vulnerabilities, including potential data disclosure and unnecessary processing of personal data.

### 2.2 Community-led approach to secure data collection and recruitment

The recruitment strategy centred on two community co-applicants; each selected for their trusted standing and established connections within their respective communities. Co-applicants were provided with smartphones and acted as gatekeepers to recruit participants to the WhatsApp groups. The Research Assistant (RA) set up WhatsApp Business accounts on project-funded devices to support participant recruitment and engagement, as the platform enables broadcast messaging, secure one-to-one communication for consent and follow-up, and easy sharing of rich media (22). Each co-applicant contributed a list of 25 men from their community networks who met the study's inclusion criteria and had given permission for their anonymised phone number to be shared for potential inclusion in the study (total *N* = 50). One WhatsApp group was created for each community co-applicant, with each group serving two interconnected functions: facilitating the intervention and dissemination of study materials and enabling secure consent and survey data collection. The sample size for this pilot was guided by the co-applicants, based on how many men they felt could realistically be recruited through convenience sampling within their existing community networks.

Inclusion criteria required participants to be men aged 45 years and older, of Somali or African-Caribbean heritage, to belong to the same community and known to the co-applicant leading the group, and to have access to WhatsApp. Contact details for eligible participants were stored securely, and the RA supported the community co-applicants in establishing and managing WhatsApp groups for study dissemination. This approach was informed by the co-applicants’ insights that WhatsApp was already widely used within their communities and regarded as a safe and trusted space for sharing health information (16). After the data collection phase, the research team became aware that one co-applicant had established a separate WhatsApp group for twelve individuals who had initially joined the main group but had subsequently left because they did not feel comfortable participating alongside people with whom they were unfamiliar. The separate group was managed by the co-applicant and provided an alternative channel for dissemination of study information. Of these twelve individuals, eight subsequently participated in the research. As these individuals had originally been recruited to the main WhatsApp group and had already been issued a study access code, their participation in the separate group did not alter the established recruitment or participation verification process. No research data or WhatsApp conversation data were collected from either group; researchers had access only to the study materials disseminated through the groups, and participant identifiers were removed. These insights were fundamental to shaping the intervention, leading the research team to prioritise a method that aligned with community expectations of acceptability while meeting ethical requirements for secure, culturally appropriate, and safe participation.

### 2.3 Dissemination of study materials

The two community co-applicants led the dissemination of study materials within the WhatsApp groups. These materials included peer-developed PCa risk messages (Figure 1), a link to the study survey, and a link to the Prostate Cancer UK (PCUK) online risk checker, which was used as an indicative measure of behavioural response following exposure to the messages. Dissemination occurred on two occasions: first during an in-person meeting with the Principal Investigator (PI), the RA, and the co-applicant from the Black African-Caribbean community, and subsequently during a meeting with the co-applicant from the Somali community. During these meetings, each co-applicant shared a series of introductory messages and PCa risk messages within their WhatsApp groups, followed by the survey and risk checker links. Two weeks later, these materials were reposted to support ongoing engagement.

### 2.4 Digital system workflow (landing page, verification, language selection, consent)

Participants accessed the study surveys through a landing page developed in collaboration with a team of Computer Science researchers (SL, GT, YL, & XW). On the landing page, participants entered their mobile number and completed a CAPTCHA to verify they were human. Once verified, a secure access code was sent via TextGlobal, a bulk SMS service procured and configured by the PI and RA. Initially, UK and Somali dialling codes were offered, but all participants had UK numbers, thus authentication was restricted to simplify access and mitigate malicious requests. After receiving the access code, participants were directed to a language selection screen offering English and Somali options. This choice determined which culturally and linguistically adapted participant information sheet and consent form was presented, with both materials refined after being piloted by the African-Caribbean and Somali groups.

### 2.5 Consent process and data handling

To participate, men were required to tick all consent boxes, enter their name, and provide a valid date. Once consent was given, completed consent forms containing personally identifiable information were encrypted using a secure software-based encryption process (Python Encryption Libraries). Keys were generated using a cryptographically secure random generator; the master keys are stored in a KSM, and data-encryption keys were protected through envelope encryption. Specific technical details of the encryption implementation, including the encryption algorithms, key length, and key-management processes, cannot be disclosed for security reasons. To support project oversight, the Computer Science researchers created a consent management interface, allowing the RA to monitor submitted consent forms alongside their associated access codes. Personally identifiable information, such as names, were removed from research datasets after verification, and participants were assigned unique identifiers (PIDs) to enable pseudonymised linkage of their responses.

### 2.6 Survey access, verification, and data management

After completing the consent process, participants were automatically redirected to an evaluative survey, hosted on Microsoft Office Forms. Multiple security steps ensured that only invited participants could access the survey. The RA kept a password-protected record of mobile numbers, access codes, submitted consent forms, and survey completion times. The mobile numbers were hashed (encrypted so they could not be used without permission) to prevent unauthorised use. This system also helped track responses accurately and identify any attempts to access the survey from outside the designated WhatsApp groups.

During early testing, the team identified potential security risks, leading to the implementation of a double-verification system whereby participants had to be pre-authorised by mobile number and then required to use a unique access code. Mobile numbers from the original WhatsApp group were securely stored, and numbers not on the approved list were automatically blocked from requesting a code. To minimise repeated attempts, participants were initially limited to five code requests, later reduced to three. During testing, a small number of attempts were made to submit data from phone numbers not registered in the WhatsApp groups, involving three IP addresses. Although it was not possible to determine whether these represented distinct individuals or multiple attempts by the same person, the requests were identified by the technical team and reported to the wider project team. Inspection suggested that the activity was limited to reconnaissance, with no evidence that the system had been successfully exploited. The event was therefore not referred to the University's central IT teams.

The final digital system, shaped by participant feedback and iterative development, supported secure, community-focused data collection while safeguarding data integrity. Although efforts were made to minimise digital exclusion, reflections on evaluation data indicate that some barriers likely persisted. This point is revisited in the discussion. Building on these practical considerations, the next section outlines the technical architecture that underpinned these protections, explaining how the system was designed to maintain data security throughout the entire recruitment and survey process.

### 2.7 System architecture and data security framework

Information Assurance is a cybersecurity concept that focuses on protecting information at every stage of a process (see Figure 2). Its core aim is to ensure that data remains confidential, accurate and unaltered (integrity), and available when needed, often referred to as the CIA principles (23). It also incorporates authenticity and non-repudiation, ensuring that data originates from a trusted source that actions cannot be falsely denied.

**Figure 2:**
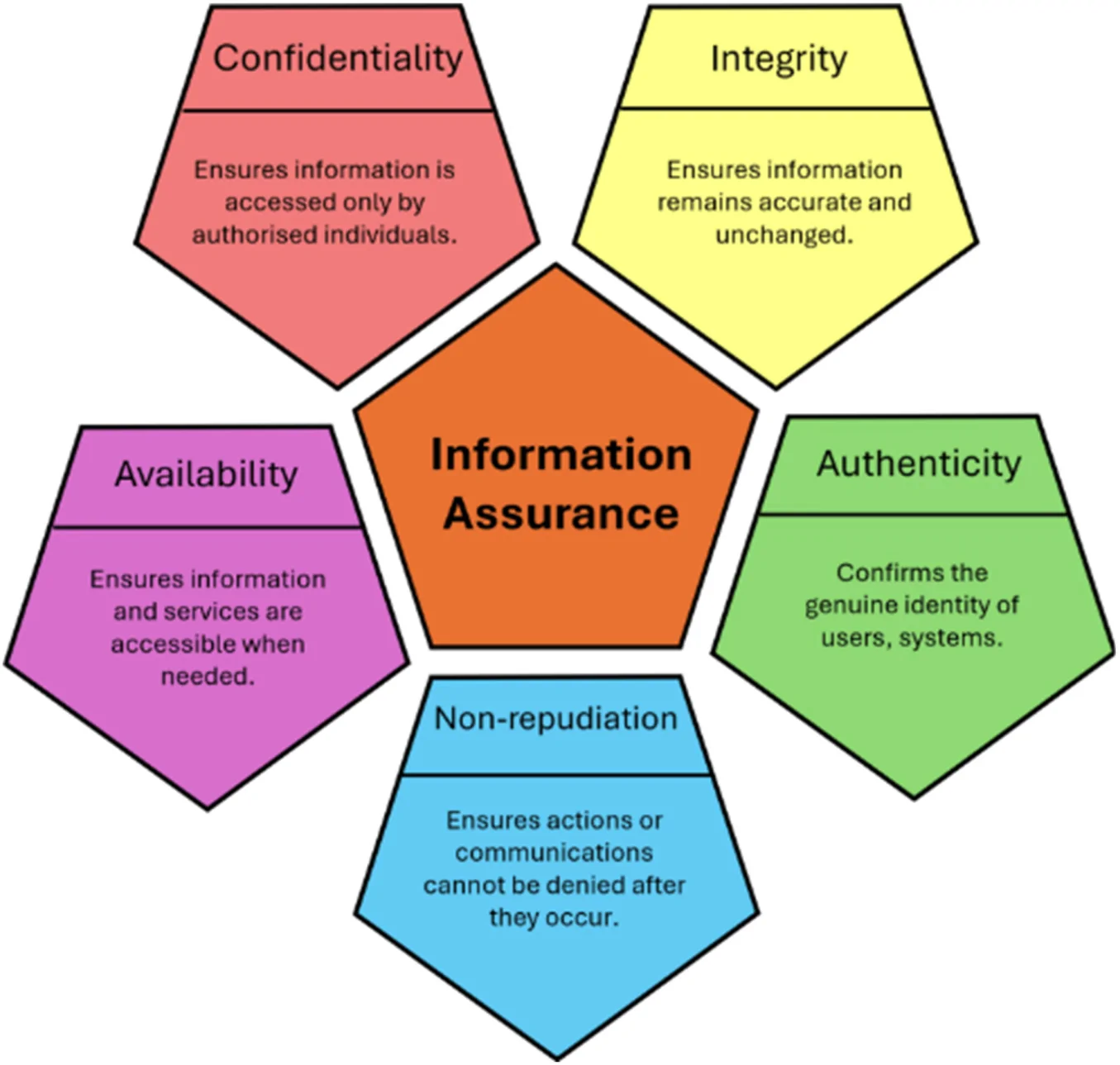
Information assurance framework. This framework illustrates the core principles of confidentiality, integrity, and availability applies to participant data protection.

To protect participant data throughout recruitment and data collection, we operationalised the CIA (Confidentiality-Integrity-Availability) security principles by developing a multi-layered secure online survey framework (see Figure 3). This framework combined encrypted communication, integrity through controlled authentication, and availability through compliant and resilient data storage practices.

**Figure 3:**
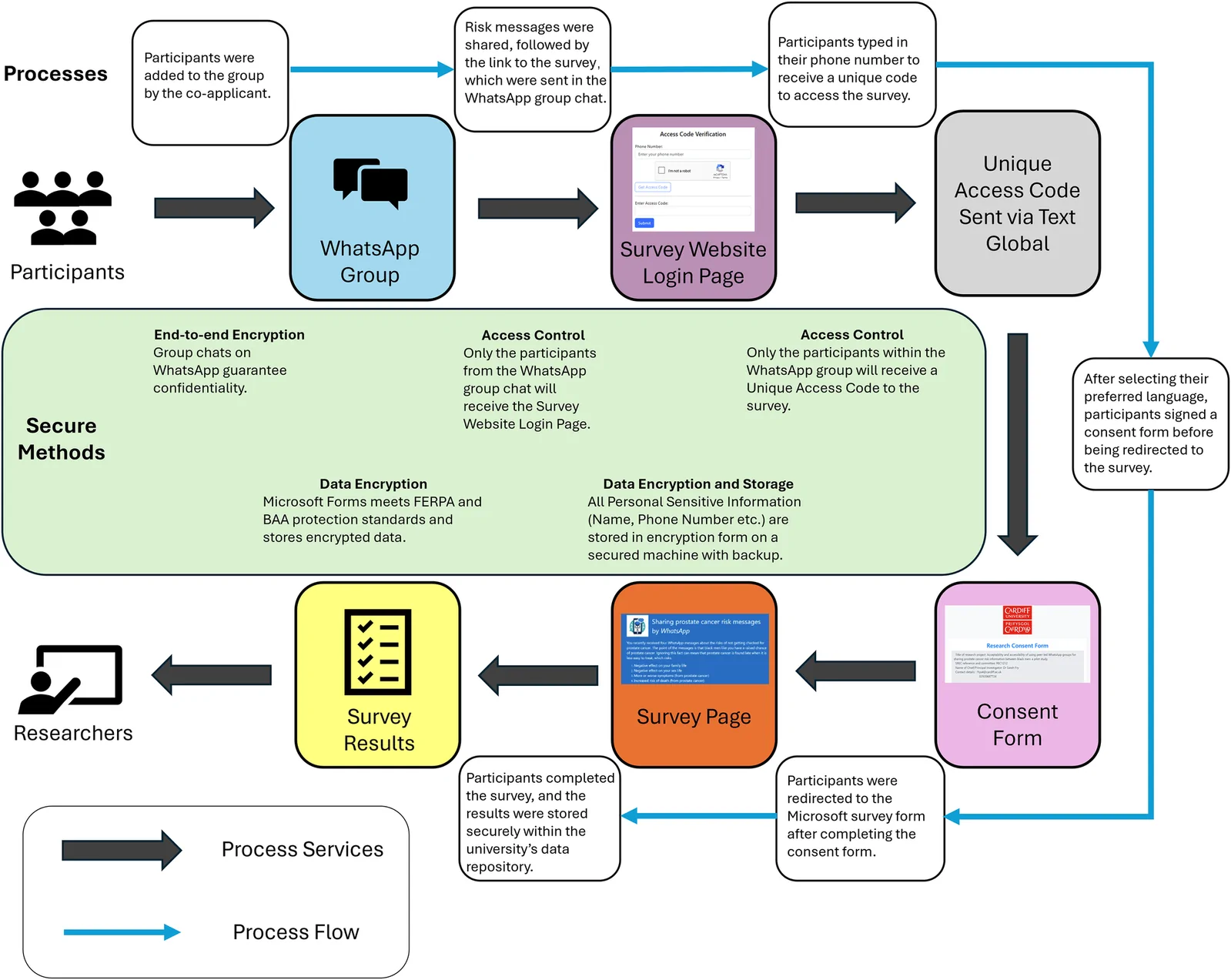
Secure online survey framework. Schematic representation of the digital system developed for community-based PCa research, showing recruitment and dissemination via WhatsApp, mobile number verification, consent collection, language selection, encryption, and secure data storage.

### Access control

Recruitment began through WhatsApp groups, which acted as the main communication and notification channel. WhatsApp's end-to-end encryption provided an initial layer of protection. Access to the survey and consent environment was controlled separately through participation authentication and mobile-number verification

### Data encryption and storage

After authentication, participants selected their preferred language and completed an electronic consent form hosted on the university's secure server. The consent process complied with ethical and regulatory standards and incorporated Python-based encryption to protect personally identifiable information. Once consent was obtained, participants were redirected to the survey on Microsoft Forms, which provides encryption in transit and at rest under institutional data governance agreements.

Personally identifiable information (names, phone numbers, consent forms) were encrypted and stored on the university server with routine backups. Rather than storing full copies of the consent forms as static files, only the essential information was saved. When a consent form was needed, the system automatically generated the PDF from the stored data. This approach maintained data resilience while upholding privacy-by-design principles (24).

The framework in Figure 3 illustrates a secure, scalable, and participant-centred method for conducting digital health research. By combining accessible communication platforms with robust security protocols, the system should promote participant trust, data protection, and compliance with institutional and ethical standards.

Following system development, we conducted a structured privacy review using the LINDDUN GO privacy threat modelling framework to assess potential risks to personal data (25). The review addressed the seven LINDDUN threat categories, including Linkability, Identifiability, Non-repudiation, Detectability, Disclosure of information, Unawareness, and Non-Compliance, ensuring that privacy considerations were embedded across the system design. An independent security review or penetration test would provide an additional assessment of the system's security; however, this was beyond the scope of the current feasibility study. The system was developed through a small grant, and the available budget was not sufficient to support associated costs. Independent security testing is therefore identified as an area of future work.

### 2.8 Privacy threat modelling using LINDDUN GO

LINDDUN GO is a structured privacy threat modelling framework that supports the systematic identification of privacy risks in digital systems (21). Using category-specific prompts presented as cards within the LINDDUN GO deck, the framework guides researchers to examine how personal information could be exposed, misused, or otherwise compromised, and to develop appropriate mitigation strategies. This approach enabled a comprehensive assessment of privacy risks and informed the implementation of safeguards aligned with ethical and regulatory requirements. There are two other variants of the LINDDUN methodology that exist, including LINDDUN PRO and LINDDUN MAESTRO. However, the PRO variant is unsuitable for our system, as we do not implement everything from scratch. PRO analyses code-level interactions, but we do not have access to the implementation details of WhatsApp or Microsoft Survey Forms. MAESTRO is a much more recent and advanced variant (introduced in 2024) with seven architectural viewpoints that create an exhaustive view of the system being assessed. In this pilot study, we chose GO, as our system at its current stage is not complex enough to warrant the depth of coverage that MAESTRO provides. For more complex systems, or for the future versions of our system, MAESTRO is an option to consider. This choice also meant that the privacy assessment provided a proportionate assessment for the pilot system but did not provide the depth of analysis that would be possible using a more comprehensive methodology.

Below, we describe relevant threats identified through the LINDDUN GO card deck, alongside the specific mitigation strategies implemented.

D1: Inferring the existence of a user from the system's response. When submitting an email address or phone number during registration, no error messages are shown that might reveal the existence of an account associated with that information. Instead, any error messages direct users to contact the research team.

DD1 & DD2: The system collects more sensitive or granular data than necessary. Only the minimum necessary data, in terms of type, amount, and granularity, is collected from participants.

DD3: Unnecessary further processing, analysis, or enrichment of data. Data is processed solely to achieve the research aims, with no additional enrichment or secondary analysis.

DD4: Data is stored for longer than necessary. At the end of the project, data will be archived in encrypted form and permanently deleted after five years.

DD5: Unnecessary sharing of personal data with external parties. Data is encrypted when stored on OneDrive or in the University Data Repository. Unencrypted data is shared only with team members who require access for system functionality or research purposes. Survey data is initially stored on SharePoint (as per Microsoft Forms) but is then managed as described above.

U5: Participants are unable to rectify or erase their personal data. WhatsApp messages are automatically deleted after 24 h. Participants may also contact the PI to request deletion or correction of any submitted data. Once deleted, there is no way to infer any link between the participant and remaining project data.

U1: Participants are not adequately informed about data collection and processing. Participants are fully informed through the participant information sheet and the informed consent form.

Nc1 & Nc2: System non-compliance with privacy regulations or standards. The project received full approval from the University’s Ethical Review Board prior to commencement.

Lawful basis for processing special category data: Ethnicity data were collected as special category personal data and processed under UK GDPR Article 9 (2)(j) for scientific research purposes, alongside Article 6 (1)(e) as the lawful basis for processing personal data.

Nc3: Inadequate data management throughout its lifecycle. The project adheres to clearly defined rules for each phase of the data lifecycle: creation, storage, sharing, usage, archiving, and deletion.

Nc4: Security measures do not meet best practice. Website development followed security best practices, including input sanitisation and the use of standard encryption protocols.

Other threats within the categories described above, as well as other LINDDUN categories, such as Linkability and Non-repudiation, were also assessed but were not considered applicable to this study. For example, L1: Linked User Requests does not apply because the online survey involved is a one-time interaction, with no subsequent user requests. Non-repudiation was addressed through the consent process, which informed participants that information already obtained may be re-obtained if they subsequently withdrew from the study. As noted under DD5 above, this information was not shared with parties who did not require access to it. Given that researchers could already identify participants through the consent process, repudiation was not considered meaningful in relation to the research team, while other parties did not have access to participants’ identifying information.

Overall, applying the LINDDUN framework enabled the team to systematically identify potential privacy risks and implement safeguards to protect participants. This ensured that both technical measures (e.g., encryption, controlled access) and procedural protections (e.g., monitoring, verification) were in place throughout the study. These safeguards also supported ethical conduct by protecting participant data and upholding confidentiality, integrity, and responsible data management.

## 3 Methodological outcomes

### 3.1 Verification, authentication, and access control outcomes

Early testing identified potential security risks, which prompted the implementation of a double-verification system: participants were pre-authorised by mobile number and then required to use a unique access code. Additional safeguards included securely storing mobile numbers from the original WhatsApp groups to prevent misuse and automatically blocking any number not on the approved list.

Although a few unauthorised access attempts were observed during testing, the combination of technical controls and active monitoring successfully maintained system security during live data collection. These outcomes illustrate that careful design and vigilant oversight can balance participant accessibility with robust access control in community-based digital research.

### 3.2 Data security and integrity outcomes

Throughout recruitment and data collection, no breaches of participant confidentiality or data integrity occurred. Personally identifiable information was successfully separated from survey responses following verification, with unique participant identifiers enabling secure linkage without exposing identifying data within research datasets.

The system's encryption, controlled access, and data-handling procedures were effective in maintaining confidentiality, integrity, and responsible data management. Importantly, these outcomes also reflect the benefits of embedding trusted community co-applicants within the recruitment process. By combining secure technical systems with culturally and linguistically appropriate facilitation, participants could engage confidently with the survey while maintaining data protection.

## 4 Discussion and future directions

Despite the growing use of mobile health (mHealth) interventions, their acceptability among ethnic minority communities remains underexplored (18). This study contributes to an emerging body of research on how mHealth can be adapted to meet the cultural and linguistic needs of minority populations, aiming to improve relevance and engagement. In cancer research, this study builds on evidence supporting the use of digital methods to increase uptake of cancer screening (26–29) by providing the secure digital infrastructure needed to evaluate these approaches. More broadly, this paper illustrates how community engagement can be combined with a privacy-centred digital system to support community-based health research.

Using WhatsApp as a recruitment and dissemination tool provided a fast, low-cost, and familiar communication channel that leveraged existing community networks. In line with prior research suggesting that men in African-Caribbean and Somali communities may access health information through interpersonal or community-based channels (13), rather than institutional or clinical sources (7, 13, 15). Recognised sender names (e.g., “Cardiff University”) supported clarity and legitimacy, helping participants navigate the process. These design features demonstrate how culturally informed dissemination strategies, implemented through familiar platforms and trusted intermediaries, may provide a useful approach for reaching communities historically underrepresented in PCa research.

However, the use of familiar and accessible platform also required careful consideration of privacy and data security. The system also underscored the value of integrating multiple layers of verification, encryption, and secure storage to safeguard participant and research data. Early testing identified potential security risks, informing the implementation of a double-verification process and additional safeguards to support data security, integrity, and compliance with ethical requirements during live data collection. These methodological experiences illustrate how privacy-by-design approaches can be operationalised in digital health research and highlight the importance of robust data-governance structures. However, digital health frameworks are generally more prescriptive, often specifying requirements such as restrictions on cross-border storage of health data, whereas LINDDUN is primarily diagnostic, identifying potential points at which privacy protections may fail within a system. LINDDUN also differs in its focus on system engineering rather than higher-level governance requirements, such as data processing agreements or clinical safety case documentation.

Other frameworks, such as the NHS Digital Technology Assessment Criteria (NHS DTAC), are designed to assess technologies intended for integration with NHS services and were therefore not directly applicable to the system developed in this study. The system also had limitations that offer important directions for improvement. The separation between consent forms (hosted on a Python-based interface) and survey data (hosted on Microsoft Forms) required additional coding to redirect participants and added complexity to generating unique survey links. Fully integrating consent and survey data within a single Python-based platform would have required more time to implement but would offer the advantage of consolidating all study processes within one secure system. This approach could streamline workflow, reduce manual processes, improve data linkage, and strengthen digital governance, providing a more seamless participant experience while maintaining high standards of security and confidentiality. The approach could be implemented by research teams or public health organisations with appropriate technical and data-governance expertise. Although developed for a pilot study, the underlying approach could be adapted to other community-based health research contexts, with further development and evaluation required to assess its scalability across larger participant groups and different research settings.

While the system was designed to minimise digital exclusion and enhance accessibility, some barriers likely remained. Participants needed access to smartphones, WhatsApp, and sufficient digital literacy to navigate the verification and consent pathway. These factors may have influenced engagement and participation, particularly among underrepresented groups (30). Participants’ digital literacy was considered in the design and implementation of the intervention, and detailed analysis of its effects will be presented in a subsequent findings paper.

Our novel methodological and practical insights are directly relevant to the study's focus on PCa awareness and risk among Black men in Wales. By embedding secure, community-led recruitment within trusted networks, the system was used to supported timely and culturally appropriate dissemination of PCa risk messages and the Prostate Cancer UK (PCUK) risk-checker addressing known barriers to awareness and early diagnosis while the UK continues to lack universal screening options (11). Consequently, the lessons learned from the development and implementation of this digital system have broader implications for designing inclusive, secure, and culturally responsive health-communication interventions for populations in which trust, representation, and community leadership are critical. Additionally, the secure digital infrastructure presented in this paper provides guidance for researchers using WhatsApp in mHealth research, highlighting how ethical and governance frameworks can be used to support secure, privacy-conscious data collection (18).

### 4.1 Strengths and limitations

A key strength of this study was the integration of accessible community involvement with privacy-centred digital system development. Community co-applicants informed the choice and use of WhatsApp as a familiar communication platform, while the technical infrastructure incorporated layered authentication, encryption, controlled access, and privacy threat modelling using LINDDUN GO. The iterative development process also allowed security risks identified during early testing to inform additional safeguards before live data collection.

Several limitations should also be considered. This was a small, community-based pilot using convenience sampling through established community networks, and the approach may therefore not be directly transferable to populations or settings without similar community infrastructure. As this study focused on the development and implementation of the digital system, it did not generate outcome data that would allow the feasibility, engagement, or impact of the approach to be assessed directly. Participation required access to a smartphone, WhatsApp, and sufficient digital literacy to navigate the authentication and consent process. Digital access and literacy remain barriers for some participants, particularly in settings where equivalent support is not available. The system relied on several interconnected platforms and services, including WhatsApp, a Python-based consent interface, TextGlobal, Microsoft Forms, and university data storage, which introduced additional technical and governance complexity. Finally, while LINDDUN GO provided a structured privacy threat assessment, the system did not undergo independent penetration testing or security review.

## Data Availability

The original contributions presented in the study are included in the article/Supplementary Material, further inquiries can be directed to the corresponding authors.
